# A Novel Multimedia Player for International Standard—JPEG Snack

**DOI:** 10.3390/jimaging9030058

**Published:** 2023-03-01

**Authors:** Sonain Jamil, Oh-Jin Kwon, Jinhee Lee, Faiz Ullah, Yaseen, Afnan

**Affiliations:** Department of Electronics Engineering, Sejong University, Seoul 05006, Republic of Korea

**Keywords:** multimedia, JPEG Snack, international standard, multimedia player, short content

## Abstract

The advancement in mobile communication and technologies has led to the usage of short-form digital content increasing daily. This short-form content is mainly based on images that urged the joint photographic experts’ group (JPEG) to introduce a novel international standard, JPEG Snack (International Organization for Standardization (ISO)/ International Electrotechnical Commission (IEC) IS, 19566-8). In JPEG Snack, the multimedia content is embedded into a main background JPEG file, and the resulting JPEG Snack file is saved and transmitted as a .jpg file. If someone does not have a JPEG Snack Player, their device decoder will treat it as a JPEG file and display a background image only. As the standard has been proposed recently, the JPEG Snack Player is needed. In this article, we present a methodology to develop JPEG Snack Player. JPEG Snack Player uses a JPEG Snack decoder and renders media objects on the background JPEG file according to the instructions in the JPEG Snack file. We also present some results and computational complexity metrics for the JPEG Snack Player.

## 1. Introduction

Recent advances in mobile communications have increased the consumption of short-form digital content based on images [1]. As the number of smartphone users has grown exponentially, it is expected to surpass $7.5\times 10^{9}$ by 2026 [2]. In [3], Koetsier reported that digital content consumption doubled in 2020. Social media platforms such as Facebook and YouTube have taken advantage of this tremendous increase in consumption by introducing Facebook Reels and YouTube Shorts simultaneously [4]. Earlier than YouTube Reels, China introduced TikTok, which quickly became popular and attracted millions of users [5,6]. In addition to uploading their short videos, users can also view other users’ videos on TikTok.

Snack culture is similar to snacks in that they can be enjoyed for a short time as a snack at any time. Snack culture was first coined in South Korea to a tremendous effect [7]. Short-form content largely replaces long-form content as an indicator of snack culture [8]. In addition to being the source of entertainment and recreation, short videos play an essential role in the education [9,10], and health industries [11,12] as well. Short videos also promote food and beverages [13]. As a result of the numerous advantages and excessive consumption of short-form content, the joint photographic experts’ group (JPEG) experts developed an image-rich content-based international standard called JPEG Snack [14].

JPEG Snack is the novel standard for representing and saving multimedia files in the .jpg format [15]. In JPEG Snack, short videos, audio, group of images, captions, and icons are embedded in the default (background) JPEG-1 file [16,17,18,19]. JPEG Snack has the following use cases: (a) Narrated photos; (b) Captioned photos; (c) Flick photos; (d) Photo slide; (e) Presentation recording [20]. JPEG Snack is Part 8 of the JPEG Systems. The last parts, named JPEG privacy and security [21], JPEG-360 [22], and JPEG linked media format (JLINK) [23], focus on the image media only. In contrast, JPEG Snack deals with multimedia objects. As JPEG Snack is the novel international standard for short content, a novel multimedia player is necessary to play the JPEG Snack file.

This article proposes a novel multimedia player for JPEG Snack. We also present several experimental results to show the results of the JPEG Snack Player. The significant contributions of the article are listed:

- Description of JPEG Snack encoded file;
- Description of JPEG Snack decoder;
- Description of JPEG Snack System decoder;
- Development of novel multimedia player for JPEG Snack files as there is no multimedia player for the JPEG Snack standard, which is in the international standard under publication stage;
- Analysis of the complexity of the player.

The rest of the article is organized as follows: Section 2 summarizes the related work. Similarly, Section 3 briefly explains the JPEG Snack encoded file, followed by Section 4, which comprehensively discusses the JPEG Snack Player. In Section 5, the experimental results are presented. Section 6 presents the comparison of the features of the JPEG Snack Player with other media players. Section 7 presents the limitations and future directions. Finally, Section 8 concludes the work.

## 2. Related Work

Multimedia usage is increasing daily [24]. The excessive use of multimedia has allowed vendors to develop multimedia standards and players. These standards include High-Efficiency Image File Format (HEIF) [25], Moving Picture Experts Group (MPEG)-4 [26], High-Efficiency Video Coding (HEVC) [27], and Versatile Video Coding (VVC) [28]. MPEG-4, HEVC, and VVC are the standards for encoding video files, whereas HEIF is a container used to store image sequences.

For instance, several multimedia players have been developed, such as VLC [29]. VLC is a cross-platform multimedia player, and it is free and open-source. It can play most multimedia files, such as videos encoded by the above-mentioned video coding standards. However, VLC cannot play a JPEG Snack file as it is saved in .jpg.

On the other hand, most image viewers can display the JPEG Snack file’s default (background) image due to backward compatibility with the JPEG-1 standard [30]. Still, the animated effects and embedded media in JPEG Snack files cannot be enjoyed.

The JPEG Snack Player application is developed by considering the limitations of the existing media players. To the best of our knowledge, this is the first media player to play a JPEG Snack file, as the standard has recently been accepted. This media player provides an opportunity for the academic research community to understand JPEG Snack by experiencing the playback representation of JPEG Snack files. This article also brings the attention of the academic research community to the newly accepted international standard JPEG Snack for storing multimedia content.

## 3. JPEG Snack Encoded File

**Figure 1:**
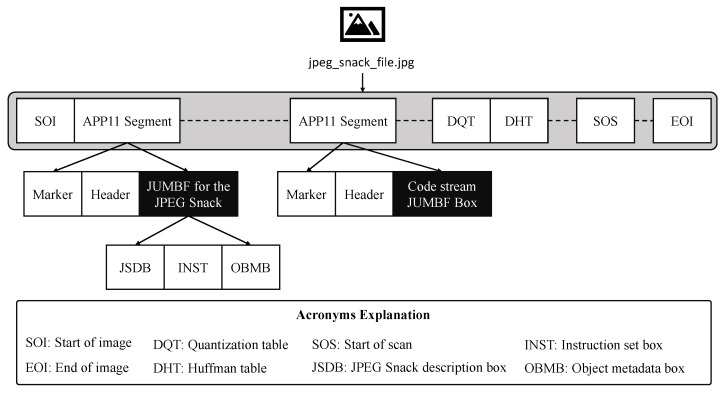
JPEG Snack file organization. Joint photographic experts’ group (JPEG) Snack file having application 11 (APP11) marker and JPEG universal metadata box format (JUMBF) boxes.

According to the ISO/IEC International Standard (IS), 19566-8 [14], a JPEG Snack file follows the ISO/1EC 10918-1 file format. In the JPEG Snack file, the application 11 (APP11) marker for the JPEG universal metadata box format (JUMBF) box [31] of the JPEG Snack representation and metadata are placed after the start of the image (SOI) marker. At the same time, the APP11 markers for embedding the media data can be placed anywhere before the start of the scan (SOS) marker. Figure 1 shows the file organization of the JPEG Snack file.

### 3.1. JUMBF Box for JPEG Snack

A JUMBF box for JPEG Snack consists of one JPEG Snack description box (JSDB), one instruction set box (INST), and multiple object metadata boxes (OBMBs).

#### 3.1.1. JSDB

A JSDB contains the number of objects and the start time required for the JPEG Snack representation.

#### 3.1.2. INST

An INST contains the information and instructions about the representation of the JPEG Snack composition.

#### 3.1.3. OBMB

Each OBMB contains the media type associated with each media object embedded in the JPEG Snack file. These media types are listed in Table 1.

These boxes are explained in detail in [14].

### 3.2. Role of Sensors

**Figure 2:**
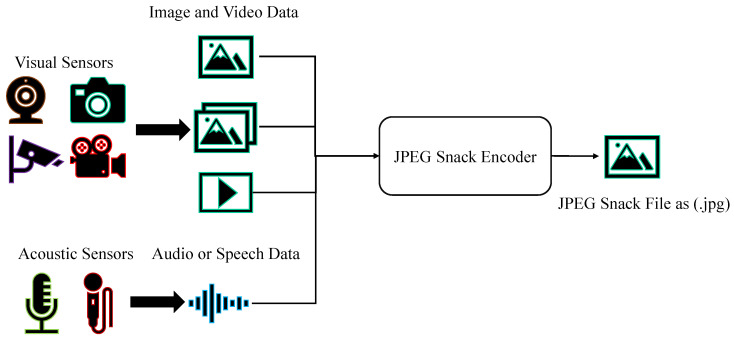
Role of sensors in the JPEG Snack file.

A JPEG Snack file needs data from visual sensors, such as the camera, and sound sensors, such as the microphone. As explained above, the JPEG Snack file contains embedded multimedia data, so the inputs to the JPEG Snack encoder can be portable network graphics (PNG) or JPEG-1 images taken from the camera, videos recorded with the camera, or audio recorded with a microphone. The sensors used for the images and videos are compact cameras [32], 360° cameras [33], digital single-lens reflex (DSLR), and adventure cameras. For audio, microphone sensors are used. The role of sensors in the JPEG Snack file is illustrated in Figure 2.

## 4. Methodology

**Figure 3:**
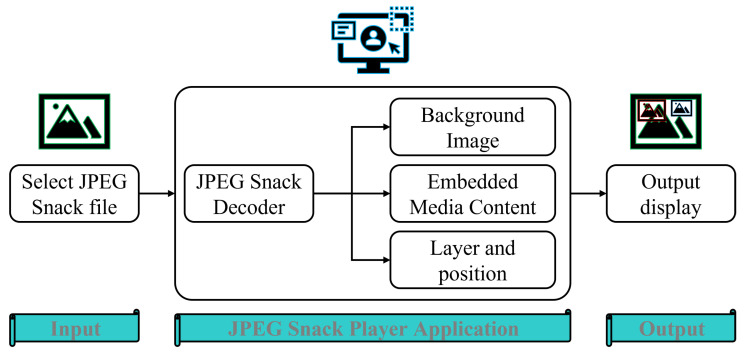
High-level flow diagram of the JPEG Snack Player.

The backbone of the JPEG Snack Player is the JPEG Snack decoder. The JPEG Snack decoder decodes the JPEG Snack file, and the decoded information is rendered. The JPEG Snack Player displays JPEG Snack representations based on the layer and position information obtained from the JPEG Snack decoder. The high-level flow diagram of the JPEG Snack decoder is shown in Figure 3.

### 4.1. JPEG Snack Decoder

**Figure 4:**
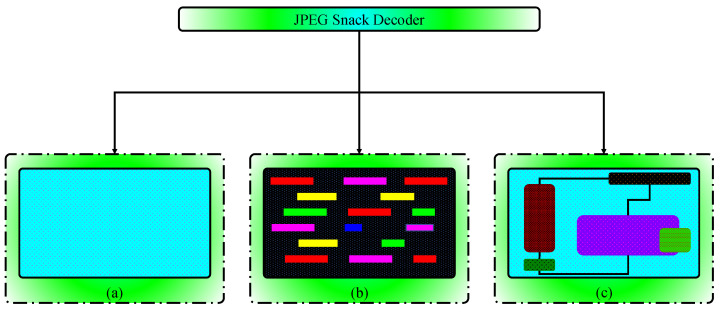
Components of JPEG Snack Decoder, (**a**) Default image (**b**) Timeline (**c**) Layer and position.

Three things are required to decode the JPEG Snack: (a) the background default JPEG image, (b) the playback timeline, and (c) the layer and position of the snack. These components are shown in Figure 4. JPEG Snack decoders decode default background images and translate instructions about displaying embedded objects on the default images. The default image is a JPEG-1 background image with JPEG Snack content embedded using APP11 markers. An embedded object’s timeline tells when it will appear on the background image and for how long. The layer and position of the embedded object specify on what portion of the default image it will be displayed and what its size will be.

### 4.2. JPEG Snack System Decoder

**Figure 5:**
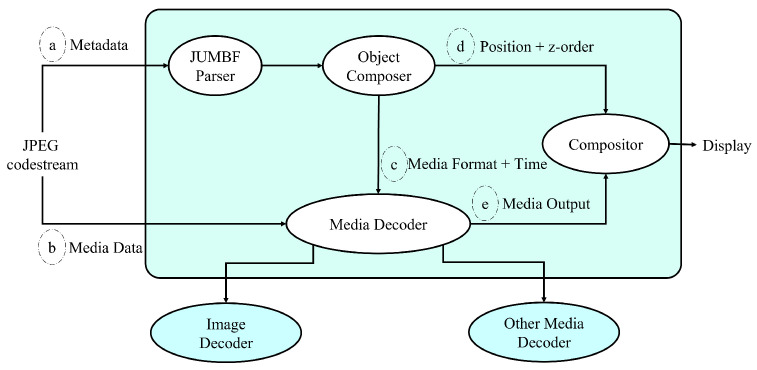
Components of JPEG Snack System Decoder.

A JUMBF parser delivers the JPEG Snack stream to the system decoder. JPEG Snack streams contain media and metadata about object structures and composition descriptions. The appropriate media decoders are invoked, and compositor-object descriptions control playback on the local device. The JPEG Snack system decoder is shown in Figure 5.

JPEG Snack’s system decoder takes JPEG codestream data. There are two types of embedded JUMBF boxes: JPEG Snack content type JUMBF boxes and embedded file content type JUMBF boxes. Metadata are in the JPEG Snack content type JUMBF box, whereas media data are in the embedded file type JUMBF box. A JUMBF parser extracts metadata and passes them through an object composer. From the JUMBF parser output, the object composer extracts media format, time, and position. The media decoder takes inputs such as media format, time, and media data and outputs media files. Media decoders can decode images or other media formats. Media output and z-order from the object composer are sent to the compositor, which creates snack representations and displays them according to playback timelines.

### 4.3. JPEG Snack Player Algorithm

**Figure 6:**
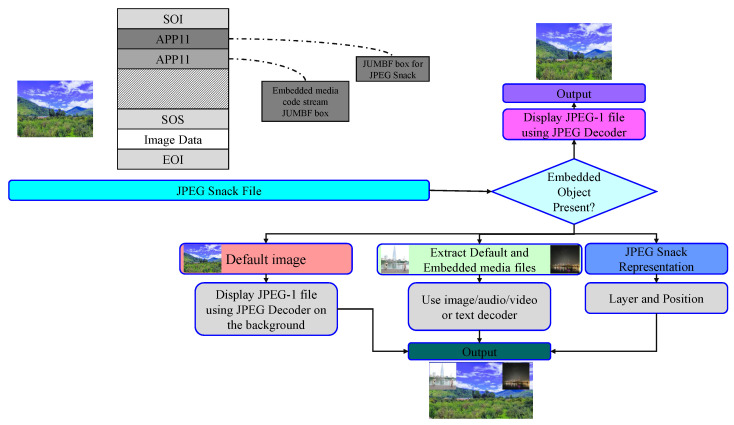
Visual representation of the steps involved in the JPEG Snack Player Algorithm.

JPEG Snack Player follows Algorithm 1. Initially, the JPEG Snack file is decoded, and after decoding, the background JPEG image is picked from the media files and displayed on the player’s screen. After showing the background image, the embedded media files are displayed according to the layer and position information related to each media file. The embedded media files are audio, videos, captions, and a group of images. The media type tells us about the embedded media; if it is audio, then the audio player is used to play the audio concurrently with the background image and the time specified in the JPEG Snack file. If the embedded file media type is an image, it is displayed in the background image according to the position specified in the JPEG Snack file. Similarly, if the embedded file media type is video, then a video player is used to play video in the specific position of the background JPEG image. Captions are also overlaid on the background image according to the information in the JPEG Snack file. Figure 6 shows the visual representation of the steps involved in the JPEG Snack Player Algorithm.
**Algorithm 1:** JPEG Snack Player Algorithm
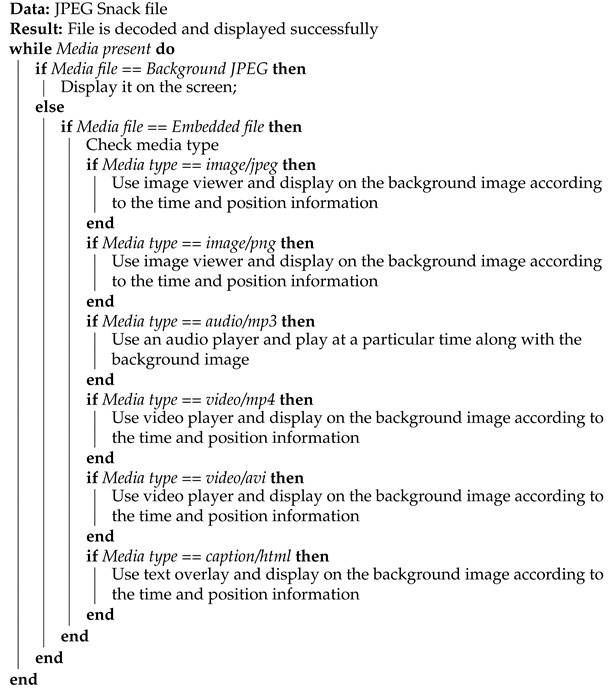


## 5. Experimental Results

The JPEG Snack Player enables users to play JPEG Snack files in three different modes:

1. Normal mode;
2. Fast mode;
3. Slow mode.

**Figure 7:**
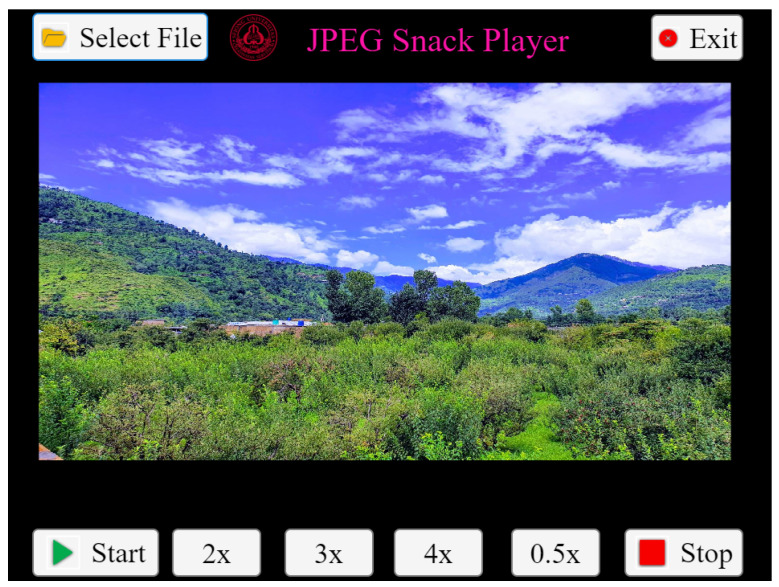
When a JPEG Snack file is selected by the user.

When the select files button is pressed, it allows the user to pick the JPEG Snack file with two objects embedded in it with the following values of the JSDB, INST, and OBMBs described in Appendix A in Table A1, Table A2, Table A3 and Table A4. When the file is selected, the background image is displayed on the plot area of the player, as shown in Figure 7.

**Figure 8:**
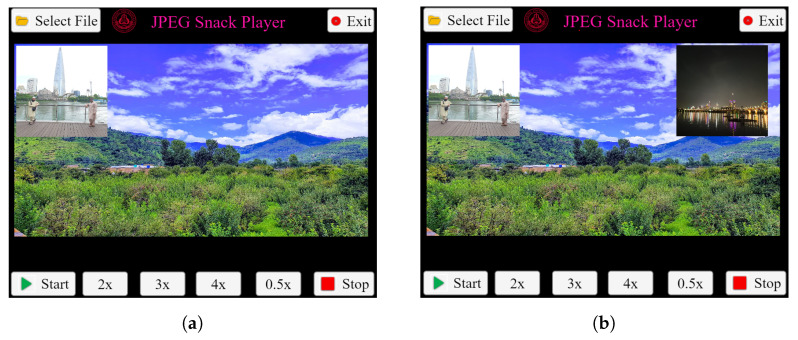
JPEG Snack Player playing JPEG Snack file with 2 JPEG Objects embedded in it. (**a**) Object 1 is displayed after 2 s. (**b**) After 3 s, Object 2 appears.

As in this JPEG Snack file, two objects are embedded, so these two objects are displayed according to the instructions. The first object is displayed after two seconds, as the start time is 2000 ms, and is shown in Figure 8a. Object 1 persists, and the second object is displayed after three seconds, as shown in Figure 8b.

**Figure 9:**
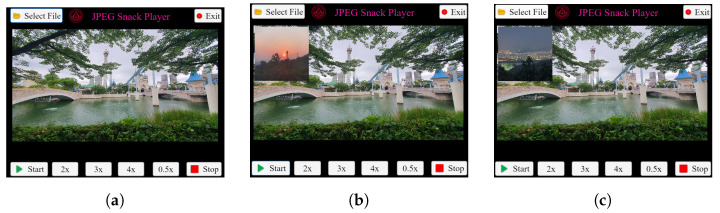
JPEG Snack Player playing JPEG Snack file with a sequence of images. (**a**) When the JPEG Snack file is selected, the JPEG-1 image is displayed. (**b**) After 2 s, the first image from the sequence is displayed. (**c**) After 3 s, the second image from the sequence is displayed.

JPEG Snack files can have embedded images, audio, videos, a group of images, and captions. Therefore, JPEG Snack Player can play all the multimedia mentioned above on the background JPEG image. Figure 9 shows the JPEG Snack player playing a JPEG Snack file in which a group of photos is embedded. The values of JSDB, INST, and OBMB are presented in Appendix B in Table A5, Table A6 and Table A7, respectively. In this example, when the JPEG Snack file is selected, the background JPEG image is displayed, as shown in Figure 9a. When the JPEG Snack file is played, after two seconds, the first image from the sequence of images is displayed as shown in Figure 9b. Similarly, after three seconds, the second image from the series of images is shown on the JPEG Snack Player, as shown in Figure 9c. After four seconds, all the pictures disappear.

**Figure 10:**
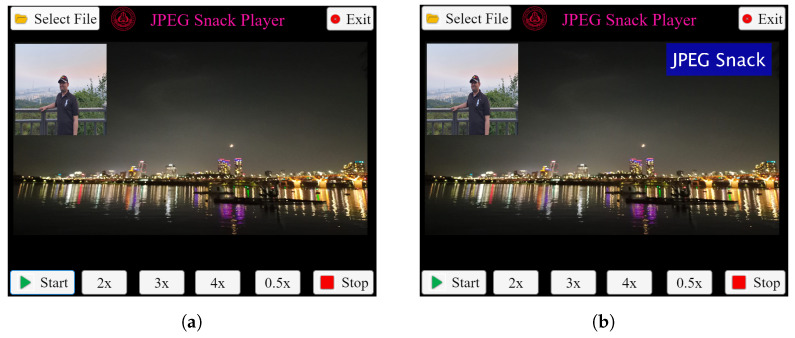
JPEG Snack Player playing JPEG Snack file with 1 JPEG image and caption embedded in it. (**a**) After 2 s, Object 1 appears. (**b**) After 3 s, the caption appears.

Similarly, Figure 10 shows the JPEG Snack Player playing a JPEG Snack file with a caption and JPEG image embedded. In this example, a JPEG-1 image and caption are embedded in the background JPEG file. After two seconds, the first object, i.e., JPEG-1 image, appears on the background image. After three seconds, the embedded caption appears on the image. JPEG Snack Player extracts the media type of the embedded multimedia files and plays accordingly. The values of JSDB, INST, OBMB for Object 1 and OBMB for Object 2 are presented in Appendix C in Table A8, Table A9, Table A10 and Table A11, respectively.

**Figure 11:**
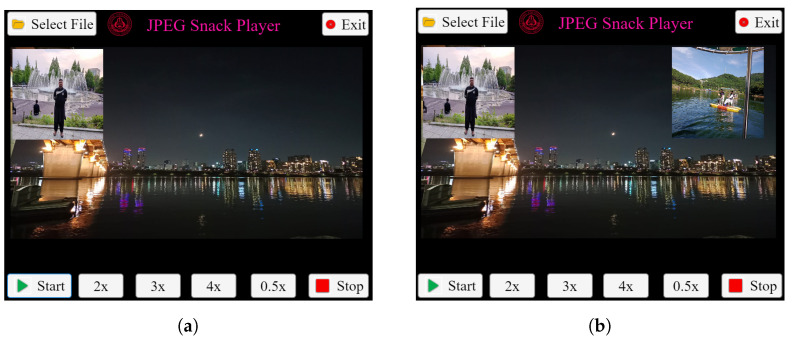
JPEG Snack Player playing a JPEG Snack file with a JPEG image and video embedded in it. (**a**) After 2 s, Object 1 appears. (**b**) After 3 s, Object 2, i.e., video, appears.

Likewise, Figure 11 shows the JPEG Snack Player playing a JPEG Snack file with an mp4 video and JPEG image embedded. In this example, a JPEG-1 image and mp4 video are embedded in the background JPEG file. After two seconds, the first object, i.e., JPEG-1 image, appears on the background image. After three seconds, the embedded video appears on the image. The embedded video is played for a short duration of time and then it disappears as the value of persistence is zero. The values of JSDB, INST, OBMB for Object 1 and OBMB for Object 2 are presented in Appendix D in Table A11, Table A12, Table A13 and Table A14, respectively.

We also evaluated the JPEG Snack Player by calculating the performance parameters. JPEG Snack Player Application takes 9.8 MB of disk space during execution. The total application installer size is 2.6 MB.

**Figure 12:**
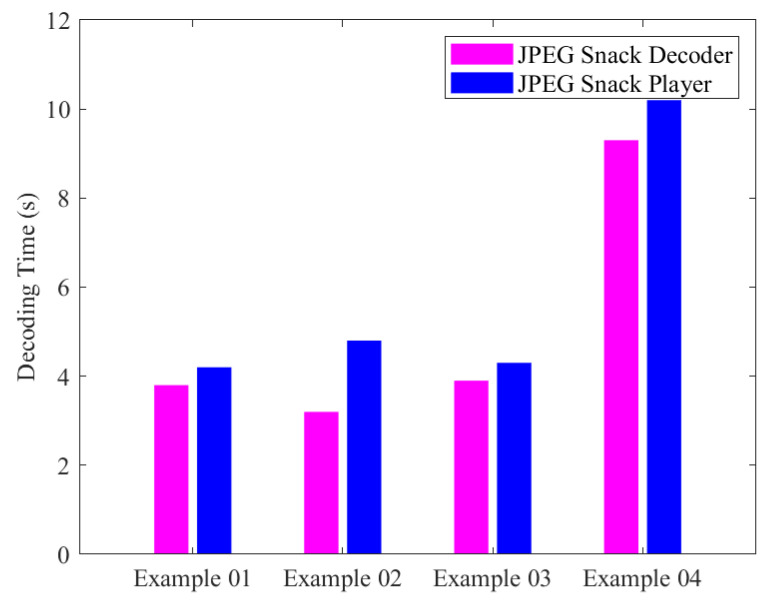
JPEG Snack Decoder and JPEG Snack Player Decoding Time.

We also evaluated the decoding time of the JPEG Snack decoder and the decoding time of the JPEG Snack Player. The following Table 2 and Figure 12 compare the decoding time in seconds. The decoding time is evaluated on a laptop having the following specifications: core-i5, 7th generation, with each core being of 2.60 GHz. The system also possesses 8 GB of random access memory (RAM). The system is also equipped with a 512 GB solid-state drive (SSD). The laptop is designed by Hewlett-Packard (HP) Computer hardware company, Palo Alto, California, United States.

## 6. Features Comparison with Other Media Players

JPEG Snack Player plays the animated short content in the JPEG Snack file, and the file is stored in the form of .jpg. The embedded media files can be displayed, and playback can be enjoyed in the video. To the best of our knowledge, this is the first multimedia player developed for the JPEG Snack standard and which supports the playback of the JPEG Snack files. In this section, we compare several features of the JPEG Snack with commonly used media players such as image viewers and VLC. All image viewers can display the JPEG-1 files, so if we open the JPEG Snack file with the image viewer, the default (background) image is displayed due to backward compatibility. Regardless, embedded media such as images, audio, video, and text cannot be viewed using image viewers.

In contrast with the image viewers, all video players support video formats such as .mp4, .avi, etc. The most widely used video player is the VLC media player, which can play most multimedia files, such as audio and videos. However, it also fails to play the JPEG Snack file. Table 3 compares the features of the JPEG Snack Player with the existing media players.

## 7. Limitations and Future Directions

Currently, the software is only available for use on personal computers in its current version. There is the possibility of extending it to a smartphone app by importing a JPEG Snack file decoder as a library in the Android application, which can be used to process JPEG Snack files. To enjoy JPEG Snack files online, the software can be extended to the web-based version to make it possible to enjoy the files online. Furthermore, it is also possible to include the JPEG Snack editor in the JPEG Snack Player so that users would be able to update and customize the embedded content of the JPEG Snack files within the player.

## 8. Conclusions

In the modern era of mobile communication, multimedia usage is increasing daily. The enormous use of image-enriched multimedia content attracted the attention of the JPEG group, leading them to propose a novel international standard called JPEG Snack. In the JPEG Snack file, the images, audio, videos, captions, and sequence of images can be encoded in the background JPEG image. The JPEG Snack file is saved and transmitted as a .jpg file. JPEG Snack needs a novel multimedia player to play the JPEG Snack content embedded in a JPEG Snack file. In this article, we proposed a novel multimedia player for playing JPEG Snack files. The JPEG Snack Player is compact and provides a platform to integrate different multimedia players such as audio, video, captions, and images that can be played on the background JPEG image in the JPEG Snack Player. The JPEG Snack Player is efficient in terms of CPU memory consumption compared to VLC and Windows Media Player.

## Figures and Tables

**Table 1:** Embedded Objects’ Media types supported by JPEG Snack standard.

Media Type	Media Format
Image	.jpeg
	.png
	.tiff
	.bmp
	.gif
Audio	.mp3
	.wav
	.flac
Video	.mp4
	.avi
Caption	.html

**Table 2:** Comparison of decoding time of JPEG Snack Player and JPEG Snack decoder.

JPEG Snack File	JPEG Snack Decoder Decoding Time (s)	JPEG Snack Player Decoding Time (s)
Example 01	3.8	4.2
Example 02	3.2	4.8
Example 03	3.9	4.3
Example 04	9.3	10.2

**Table 3:** Comparison of features of JPEG Snack player with the existing media player.

Features	Image Viewers	VLC	JPEG Snack Player
Supported Media	images	audio, video	Images and JPEG Snack files
File format	.jpg, .png, .tif, .gif	.avi, .mp3, .mp4	.jpg
Image caption display	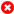	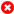	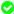
Slide show	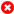	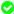 (compressed data using video codecs)	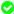 (no compression is applied to the image sequence)
Sound support	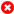	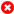	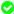
Support animation	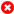	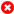	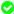

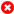
 = Unsupported 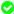 = Supported.

## Data Availability

The demo of the JPEG Snack Player can be accessed at: https://doublebench.com/2758-2/ (accessed on 8 February 2023).

## Appendix A. First Example JUMBF Boxes

Table A1, Table A2, Table A3 and Table A4 present the values of JSDB, INST, and OBMB for the first object and OBMB for the second object, respectively, for the third example.

**Table A1:** Values of the JSDB parameters for the first example.

Parameter	Value	Description
Version	1	A version of the format
Start time	2000	This denotes that after displaying the first (background image), the first object will be displayed after 2000 ms
Number of objects	2	It means that there are two media objects embedded in this JPEG Snack file
Object ID	1	This is the Object 1 identifier for the first object metadata box
Object ID	2	This is the Object 2 identifier for the second object metadata box

**Table A2:** Values of the INST parameters for the first example.

Parameter	Value	Description
Ityp	0000 0000 0000 1011	This instruction parameter shows that only the X offset (XO), Y offset (YO), width, height, persist, life, and next-use parameters are used.
Rept	1	Repetition parameter denotes that this instruction is executed once as its value is one.
Tick	1000	Tick denotes the duration of each object.
XO	10	Vertical offset of the first object.
YO	10	Horizontal offset of the first object
Width	512	Width of the first object
Height	512	Height of the first object
Persist	1	This means that the first object will be present when the second object appears
Life	0	This instruction is executed with the next instruction
Next-use	0	It means that this instruction will not be reused
XO	10	Vertical offset of the second object.
YO	1400	Horizontal offset of the second object
Width	512	Width of the second object
Height	512	Height of the second object
Persist	0	This means that the second object will not persist
Life	2	This means that Object 1 and Object 2 will be displayed simultaneously for 2 s
Next-use	0	It means that this instruction will not be reused

**Table A3:** Values of the first OBMB parameters for the first example.

Parameter	Value	Description
Toggle	0000 0000	This shows that no optional field is used
ID	1	Identifier of the box
Media type	‘image/jpg’	The embedded media is a JPEG-1 image
Location	self#jumbf = Object 1	The image is embedded in the same file

**Table A4:** Values of the second OBMB parameters for the first example.

Parameter	Value	Description
Toggle	0000 0000	This shows that no optional field is used
ID	2	Identifier of the box
Media type	‘image/jpg’	The embedded media is a JPEG-1 image
Location	self#jumbf = Object 2	The image is embedded in the same file

## Appendix B. Second Example JUMBF Boxes

Table A5, Table A6 and Table A7 present the values of JSDB, INST, and OBMB for the first object, respectively, for the second example.

**Table A5:** Values of the JSDB parameters.

Parameter	Value	Description
Version	1	A version of the format
Start time	2000	This denotes that after displaying the first (background image), the first object will be displayed after 2000 ms
Number of objects	1	It means that there is one media object, i.e., set of images embedded in this JPEG Snack file
Object ID	1	This is the Object 1 identifier for the first object metadata box

**Table A6:** Values of the INST parameters.

Parameter	Value	Description
Ityp	0000 0000 0000 1011	This instruction parameter shows that only the XO, YO, width, height, persist, life, and next-use parameters are used.
Rept	1	Repetition parameter denotes that this instruction is executed once as its value is one.
Tick	1000	Tick denotes the duration of each object.
XO	10	Vertical offset of the first object.
YO	10	Horizontal offset of the first object
Width	512	Width of the first object
Height	512	Height of the first object
Persist	0	This means that the second object will not persist
Life	0	This instruction is executed with the next instruction
Next-use	0	It means that this instruction will not be reused

**Table A7:** Values of the first OBMB parameters.

Parameter	Value	Description
Toggle	0000 0001	This shows that the number of media objects are present
ID	1	Identifier of the box
Media type	‘image/jpg’	The embedded medias are JPEG-1 images
Number of media	2	This image set consists of two JPEG-1 images
Location	self#jumbf = Object 1	The image is embedded in the same file
Location	self#jumbf = Object 2	The image is embedded in the same file

## Appendix C. Third Example JUMBF Boxes

Table A8, Table A9, Table A10 and Table A11 present the values of JSDB, INST, and OBMB for the first object and OBMB for the second object, respectively, for the third example.

**Table A8:** Values of the JSDB parameters for the third example.

Parameter	Value	Description
Version	1	A version of the format
Start time	2000	This denotes that after displaying the first (background image), the first object will be displayed after 2000 ms
Number of objects	2	It means that there are two media objects embedded in this JPEG Snack file
Object ID	1	This is the Object 1 identifier for the first object metadata box
Object ID	2	This is the Object 2 identifier for the second object metadata box

**Table A9:** Values of the INST parameters for the third example.

Parameter	Value	Description
Ityp	0000 0000 0000 1011	This instruction parameter shows that only the XO, YO, width, height, persist, life, and next-use parameters are used.
Rept	1	Repetition parameter denotes that this instruction is executed once as its value is one.
Tick	1000	Tick denotes the duration of each object.
XO	10	Vertical offset of the first object.
YO	10	Horizontal offset of the first object
Width	512	Width of the first object
Height	512	Height of the first object
Persist	1	This means that the first object will be present when the second object appears
Life	0	This instruction is executed with the next instruction
Next-use	0	It means that this instruction will not be reused
XO	10	Vertical offset of the second object.
YO	1350	Horizontal offset of the second object
Width	512	Width of the second object
Height	512	Height of the second object
Persist	0	This means that the second object will not persist
Life	2	This means that Object 1 and Object 2 will be displayed simultaneously for 2 s
Next-use	0	It means that this instruction will not be reused

**Table A10:** Values of the third OBMB parameters for the first example.

Parameter	Value	Description
Toggle	0000 0000	This shows that no optional field is used
ID	1	Identifier of the box
Media type	‘image/jpg’	The embedded media is a JPEG-1 image
Location	self#jumbf = Object 1	The image is embedded in the same file

**Table A11:** Values of the second OBMB parameters for the third example.

Parameter	Value	Description
Toggle	0000 0110	This shows that style and opacity are present
ID	2	Identifier of the box
Media type	‘text/utf-8’	The embedded media is a caption
Style	css_code	The style of the caption is embedded in the form of a style file
Opacity	0.6	The opacity of value is 0.6, which means that the transparency will be 60%
Location	self#jumbf = Object 2	The caption is embedded in the same file

## Appendix D. Fourth Example JUMBF Boxes

Table A12, Table A13, Table A14 and Table A15 present the values of JSDB, INST, and OBMB for the first object and OBMB for the second object, respectively, for the fourth example.

**Table A12:** Values of the JSDB parameters for the fourth example.

Parameter	Value	Description
Version	1	A version of the format
Start time	2000	This denotes that after displaying first (background image), the first object will be displayed after 2000 ms
Number of objects	2	It means that there are two media objects embedded in this JPEG Snack file
Object ID	1	This is the Object 1 identifier for the first object metadata box
Object ID	2	This is the Object 2 identifier for the second object metadata box

**Table A13:** Values of the INST parameters for the fourth example.

Parameter	Value	Description
Ityp	0000 0000 0000 1011	This instruction parameter shows that only the XO, YO, width, height, persist, life and next-use parameters are used.
Rept	1	Repetition parameter denotes that this instruction is executed once as its value is one.
Tick	1000	Tick denotes the duration of each object.
XO	10	Vertical offset of the first object.
YO	10	Horizontal offset of the first object
Width	512	Width of the first object
Height	512	Height of the first object
Persist	1	This means that the first object will be present when the second object will appear
Life	0	This instruction is executed with the next instruction
Next-use	0	It means that this instruction will not be reused
XO	10	Vertical offset of the second object.
YO	1400	Horizontal offset of the second object
Width	512	Width of the second object
Height	512	Height of the second object
Persist	0	This means that the second object will not persist
Life	2	This means that Object 1 and Object 2 will be displayed simultaneously for 2 s
Next-use	0	It means that this instruction will not be reused

**Table A14:** Values of the first OBMB parameters for the fourth example.

Parameter	Value	Description
Toggle	0000 0000	This shows that no optional field is used
ID	1	Identifier of the box
Media type	‘image/jpg’	The embedded media is a JPEG-1 image
Location	self#jumbf = Object 1	The image is embedded in the same file

**Table A15:** Values of the second OBMB parameters for the first example.

Parameter	Value	Description
Toggle	0000 0000	This shows that no optional field is used
ID	2	Identifier of the box
Media type	‘video/mp4’	The embedded media is an MP4 video
Location	self#jumbf = Object 2	The image is embedded in the same file
